# The Relevant Approaches for Aligning Carbon Nanotubes

**DOI:** 10.3390/mi13111863

**Published:** 2022-10-30

**Authors:** Zhifu Yin, Ao Ding, Hui Zhang, Wang Zhang

**Affiliations:** 1 State Key Laboratory for Modification of Chemical Fibers and Polymer Materials, Donghua University, Shanghai 201620, China; 2 School of Mechanical and Aerospace Engineering, Jilin University, Changchun 130000, China

**Keywords:** carbon nanotube, alignment, approach, performance improvement

## Abstract

Carbon-nanotube (CNT) is a promising material owing to its compelling mechanical, thermal and electrical properties and has been applied in a broad variety of fields such as composite, fiber, film and microelectronic. Although the introductions of CNT have brought huge improvement for many applications, these properties of macrostructures prepared by CNTs still cannot meet those of individual CNT. Disordered alignment of CNTs in the matrix results in degradation of performance and hinders further application. Nowadays, quantities of methods are being researched to realize alignments of CNTs. In this paper, we introduce the application of CNTs and review some typical pathways for vertical and horizontal alignment, including chemical vapor disposition, vertical self-assembly, external force, film assisted, electric field, magnetic field and printing. Besides that, advantages and disadvantages of specific methods are also discussed. We believe that these efforts will contribute to further understanding the nature of aligned CNT and generating more effective ideas to the relevant workers.

## 1. Introduction

Carbon nanotubes (CNTs) are seamless cylinders of one or more layer of graphene (denoted single-wall, SWNT, or multi-wall, MWNT), with open or closed ends [1]. In 1991, Iijima discovered Multi-wall carbon nanotubes (MWCNT) by high-resolution electron micrograph accidentally [2]. With that, single-wall carbon nanotubes (SWCNT) were discovered by Bethune in 1993 [3]. With the development of research, CNTs have been considered as an exciting material owing to its extraordinary intrinsic properties, such as elastic modulus approaching 1T Pa, tensile strength of 100 G Pa, electric conductivity of 10 Acm^−2^ and thermal conductivity of 3500 W m^−1^ K^−1^ [4,5,6]. To date, arc discharge [2], chemical vapor deposition (CVD) [7]. and laser ablation [8]. are the main methods for preparing CNTs. These properties are far superior to other materials, making carbon nanotubes widely used in various fields.

These fields can be grouped into the following major categories: [1,9,10,11].

1. Composite materials. CNTs are often used as additives to improve the properties of different materials, such as polymers, metals and ceramics. There are some typical examples, such as sporting goods and structural parts, which are stronger, lighter and more durable.
2. Fiber and film. After large numbers of researches, CNT fiber could become a strong competitor of carbon fiber in high-end applications due to its excellent electrical and mechanical properties [12,13,14,15,16,17,18]. CNT-based film has not only enhanced properties but transparency, which makes it seen as an alternative to indium tin oxide [19,20]. Besides that, CNTs can be used in PDMS to reduce the internal resistance and improve the output performance [21].
3. Microelectronic. In this field, due to its excellent electrical performance, quantities of application can be achieved, such as field emission [22], field-effect transistors [23,24,25], sensor [26,27,28,29,30,31,32,33], computer [34], solar cells [35]. and flexible electronics [36].

Although excellent properties have been measured for individual CNT, the macrostructures, such as yarns and sheets, remain significantly lower than the former [1], which is resulted by its highly anisotropic due to their high aspect ratio. Thus, there are two main barriers for further applications of CNTs, preventing from realizing their full potential. The first is the aggregation of CNTs, which is resulted by van der Waals attraction force between them. Generally, they are held tightly together instead of being a single nanotube. The approach, a combination of ultrasound radiation assisted by a rotational stir, was verified to be the most efficient for dispersing CNTs in epoxy [37]. At the same time, it has been proved that using functionalized CNTs can improve the uniformity of nanocomposite [38]. Secondly, the disordered CNTs exhibit a much worse performance than the ordered CNTs due to its anisotropy. For instances, the composite film with aligned CNTs records an impressive 360% improvement in conductivity in the direction parallel to the alignment as compared to the structure with randomly aligned CNTs [39]. Also, the strain sensitivity of composite film with aligned CNTs is six times higher than that with totally random CNTs [40]. Using electrospun to produce filament with aligned CNTs can lead up to 49% improvement on modulus [41]. And another important reason is that the alignment and uniformity of CNTs have a significant effect on the performance and reproducibility [42]. With respect to the alignment of CNTs, there are more methods, including electric field, magnetic field, shear force, mechanical stretch and so on. 

Taking the production process as the standard of sort, the alignment of CNTs can be divided into two classifications which are in situ (in-growth) and ex situ (post-growth) [43]. It is called in situ that alignment achieves during the process of preparing CNT. While the ex situ alignment refers to using external force, field or other techniques to align unordered CNTs which have been produced. However, when it comes to alignment of CNTs, it is widely used that vertical and horizontal alignment according to the direction of alignment which will be introduced in detail in this paper. By the way, in situ does not contain or is equivalent to vertical arrangement, as do ex situ and horizontal arrangement. For instance, combining the extrusion blown plastic film technique and floating catalyst CVD approach to produce transparent conductive films with horizontally aligned CNTs [44].

**Figure 1:**
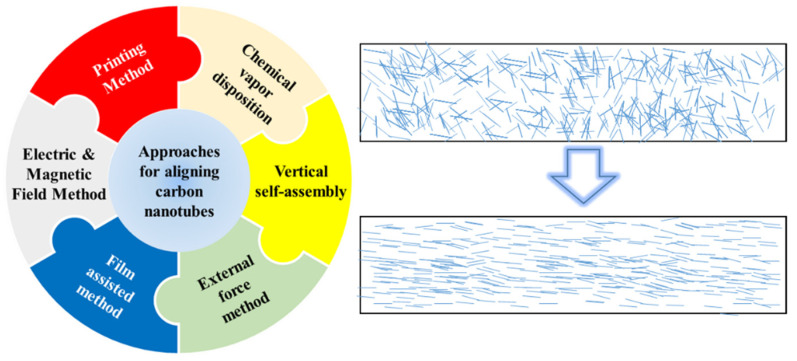
The approaches for aligning CNTs and the schematics of unordered and ordered CNTs.

Although the plethora of experiments for aligning CNTs have been done in different forms including pure CNTs, in solution and in polymer matrix, in an attempt to find some inherent regularities, we try to divide these methods into several categories on the ground of the rationales used in these approaches, as shown in Figure 1. Herein, we will give detailed introduction about these categories and their advantages and disadvantages, which can offer necessary help to these scholars who just started resarching this field. And these efforts will contribute to further understanding the nature of aligned CNT and generating more effective ideas to the relevant workers. 

## 2. Vertical Alignment Approach

### 2.1. Chemical Vapor Disposition (CVD)

CVD technique has been the most commonly used method, in particular for vertical alignment of carbon nanotube arrays (VANT) also known as “CNT forest” in which the direction of axis of CNT is normal to the substrate. In a typical CVD process, CNTs are generally believed to follow a “vapor–liquid–solid” growth mechanism [45]. Under the condition of high catalyst density, the bending of carbon nanotubes can be prevented to a certain extent, so as to realize the growth of VANT. With the development of technique, the height of VANT has increased from micrometer to millimeter and then to centimeter in the past thirty years [46,47,48]. 

**Figure 2:**
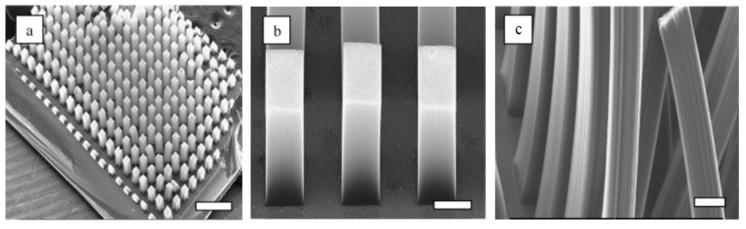
ESEM image of aligned MWCNT patterned arrays showing the effect of alignment with the grown time: (**a**) half hour, top view (scale 1 mm); (**b**) half hour, side view (scale 100 μm); (**c**) 3 h, side view (scale 100 μm) [46]. Copyright 2006, American Chemical Society.

**Figure 3:**
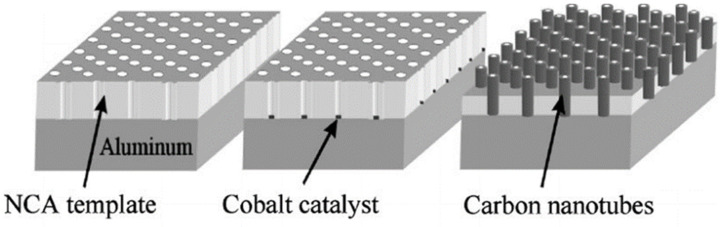
Schematic of fabrication process with a template. Reproduced with permission [49]. Copyright 1999, AIP Publishing.

Xie et al. achieved large-scale synthesis of aligned carbon nanotubes by CVD catalyzed by iron nanoparticles embedded in mesoporous silica in 1996, which is the earliest report [46]. As shown in Figure 2, using water-assisted CVD process and optimized substrate design, Yun et al. prepared the aligned VANTs with the height of 4 mm [47]. In order to avoid bending of CNTs during the growth, the template-assisted method has also become a typical method for VANTs. As shown in Figure 3, Li et al. introduced a hexagonal close-packed Nano channels alumina template to CVD for the VANT with uniform diameters and periodic arrays [49]. Using anodic aluminum oxide (AAO) template, Yuan et al. tried to find out more suitable conditions with iron as the catalyst [50]. 

### 2.2. Vertical Self-Assembly

**Figure 4:**
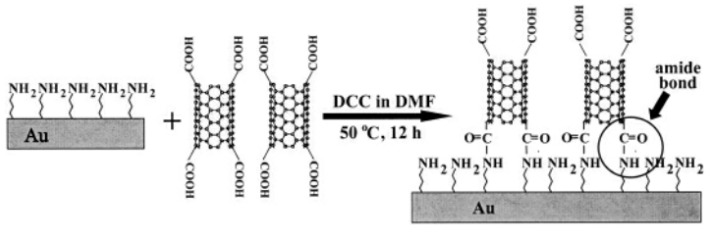
Schematic representation of the formation of SWNT assemblies. Reproduce with permission [53]. Copyright 2002, John Wiley and Sons.

Based on chelation and electrostatic interaction, Chattopadhyay et al. reported a metal-assisted self-assembly method which prepared dense arrays of SWNT and permits growth of successive stacks in a layer-by-layer assembly format [51]. By utilizing the combination of a self-assembly and a surface condensation reaction, some researchers adsorbed spontaneously the CNTs with chemical modifications of the carboxyl end groups onto different substrates of metal and silicon [52,53,54], which achieved vertical alignment of CNTs as shown in Figure 4. 

De Heer et al. proposed an approach to stretch the suspension with a 0.2-um-pore ceramic filter to produce vertical nanotube films that could be transferred onto substrates by pressing [55]. If readers want to pay more attention to VANT, there have been several articles which expound the relevant mechanism and condition of CNT synthesis in detail [45,56,57,58]. 

VANT can only be used in little applications, including field emitters, AFM tips and sensors, but need expensive equipment and precise condition, compared to horizontal aligned carbon nanotubes (HANT). It is an inherent drawback that the catalyst seen as impurity is difficult to remove nondestructively, and due to the dense arrangement, electric shielding usually brings negative effects on properties and applications of electron devices. Still, it cannot be denied that the advances of VANT techniques favor many potential applications and horizontal alignment of CNT. 

## 3. Horizontal Alignment Approach

Compared to VANT, HANT has more abundant methods. In a bid to introduce them more clearly, we divide the main approaches into several sections, including external force, film-assisted, electric field, magnetic field and printing.

### 3.1. External Force Method

#### 3.1.1. Shear Force

Shear force is a kind of common method for aligning tube and early is used to alignment of CNT. As introduced below, three classes of shear force can be distinguished: solid, liquid and gas.

(1) Solid State

**Figure 5:**
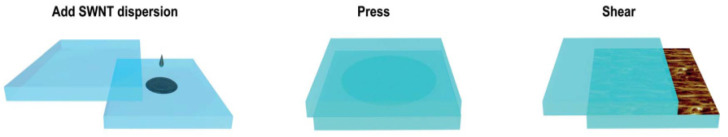
Illustration of the superacid slide casting method. Reproduced with permission [61]. Copyright 2013, Royal Society of Chemistry.

**Figure 6:**
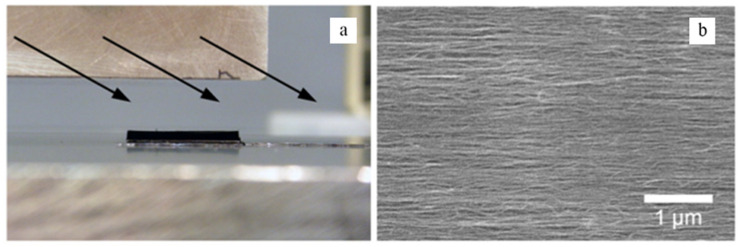
Overview of the process of shear pressing. (**a**) Process of shear pressing. (**b**) SEM image with aligned CNT after pressing. Reproduced with permission [62]. Copyright 2010, Elsevier.

A facile method was proposed by Ajayan in 1994, which provided a fresh approach for relevant researches [59]. They cut thin sheets of CNT-polymer composites and found that the CNTs were not cut off and straightened during the cutting process, thus achieving an orderly arrangement. It also was proved that shear force can align CNT in a polymer film by polarized Raman spectrum [60]. Li et al. introduced a super acid slide coating method, in which SWNTs were dissolved in chlorosulfonic acid and several droplets of this solution were sandwiched between two glass slides [61]. The slides were pressed and slid manually and then the oriented thin films of CNT were accomplished, as shown in Figure 5. Interestingly, Bradford et al. proposed a novel approach which achieved the conversation from VANT to HANT by shear pressing as shown in Figure 6.

(2) Gas State

Snow [63], Xin [64] and Hedberg [65] are foregoers in alignment of CNTs by the force of gas blow. Although they used the different setups, the forces they relied on are similar. When the solution of CNTs dropped on the Si substrate along with the gas blow, droplet dispersed in the direction of gas blow, which can achieve large-scale dispersion and alignment of CNTs easily.

**Figure 7:**
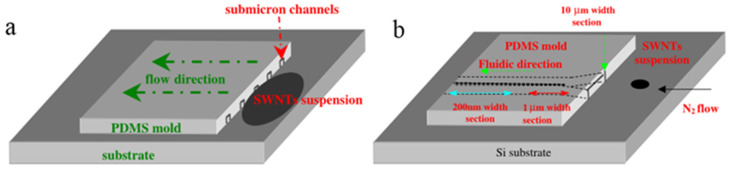
(**a**) Schematic representation of SWNT alignment by the capillary effect. Adapted with permission from [66], Copyright 2006 IOP Publishing. (**b**) improved method with funnel-shaped entrances and N_2_ flow. Reproduced with permission [67]. Copyright 2007, IOP Publishing.

Yan et al. Carried out a series of experimental studies on aligning CNT by gas shear force, which is a classic approach influent the behind. A PDMS mold with submicron channels was placed, channel side down on a substrate to form tubular channels in which the CNT droplet can align as the fluid flowed driven by the capillary effect (Figure 7a) [66]. To solve the problem of nanotubes accumulate in the channels, further researches have been done. Firstly, the substrate was slightly tilted to assist the suspension reaching the channels [66]. Furthermore, the end of the channels was designed to be the funnel-shaped microchannel entrances, and a jet of N_2_ was introduced to provide a stable gas blow for pushing the CNT solution into the microchannel (Figure 7b) [67]. Eventually, they transferred the patterns of aligned CNTs onto a functionalized electrode successfully [68].

(3) Liquid State

**Figure 8:**
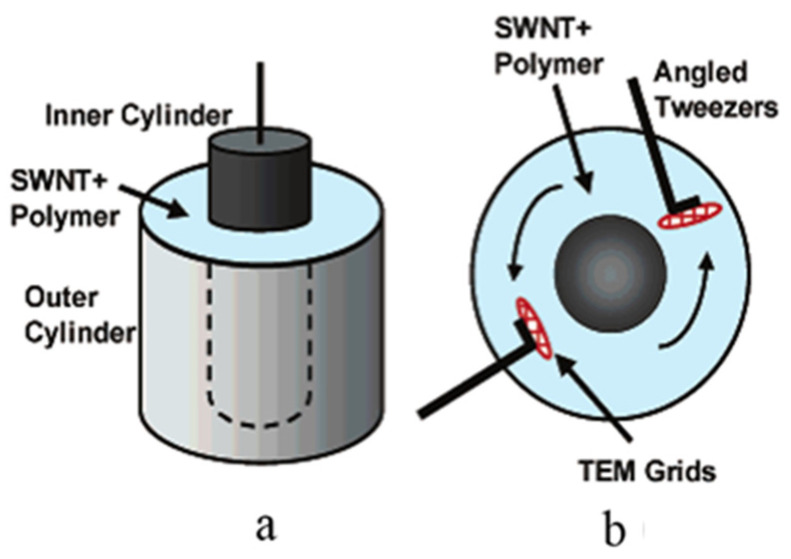
Schematic representation of the experimental setup: (**a**) Concentric cylinder arrangement in the Brookfield viscometer; (**b**) TEM sample retrieval and preparation. Reproduced with permission [70]. Copyright 2006, American Chemical Society.

Poulin et al. proposed firstly the method that aligning CNTs in liquid [69]. The SWNTs were uniformly dispersed in an aqueous solution containing surfactant SDS, and the dispersion was injected into PVA aqueous solution keeping stable stirring through a needle. The carbon nanotubes are oriented at the tip of the needle and fixed by the polymer chain segment in PVA aqueous solution. Tannenbaum et al. mixed SWNT, NaDDBS and CMC in a cylindrical container which have a rotatable inner cylinder to achieve the rotation of solution as shown in Figure 8 [70]. The results show that the addition of CMC helps to increase the dispersion and orderly arrangement of CNTS under shear force. Hobbie proposed an approach of optical measurement to describe shear-induced structure and orientation in semidilute dispersions of MWNT [71].

#### 3.1.2. Stretching Force

**Figure 9:**
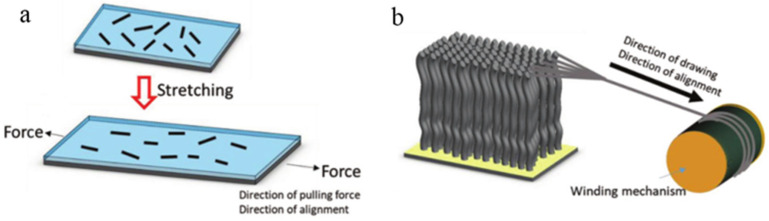
Aligning CNT by (**a**) film pulling method and (**b**) fiber drawing method. Reproduced with permission [43]. Copyright 2019, John Wiley and Sons.

In this method, thin film and fiber are main applicable objects as shown in Figure 9. Thostenson et al. created an aligned nanocomposite film of MWNT by extrusion and drawing using a rectangular die in the molten state [72]. It was found that the tensile modulus, yield strength and ultimate strength of the polymer films were improved by adding nanotubes, and the elastic modulus of the aligned CNT composites was increased 5 times than that of the randomly oriented composites. Haggenmueller et al. fabricated nanocomposite films and fibers consisting of a PMMA matrix with SWNTs by the methods of melt pressing and melt spinning [73]. Dai et al. produced the SWNT-epoxy composite by solution casting technique [74]. Subsequently, the mixture was stretched repeatedly along one direction at the semidried state for 100 times manually to achieve the alignment of CNT. Cheng et al. also employed this method in MWNT/BMI composites with high CNT concentrations [75]. The above references all showed that the conductivity and mechanical property along the flow direction was higher than perpendicular to it. Li et al. stretched pure CNT networks to align CNT and assessed the real-time degree of alignment by X-ray and Raman scattering techniques [76]. To meet various applications, Liu et al. successfully prepared tunable CNT arrays for spinning continuous unidirectional sheets [77].

**Figure 10:**
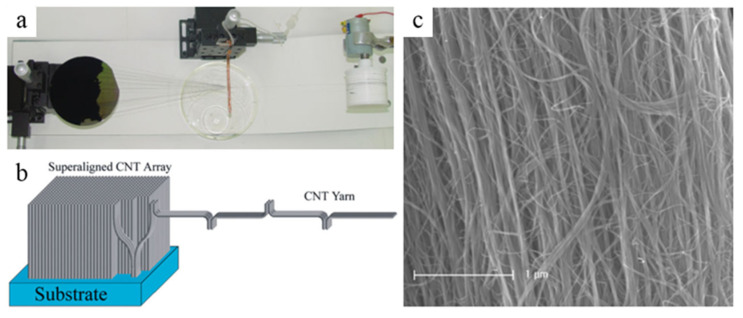
Spinning continuous yarns from super-aligned CNT arrays. (**a**) Top view of spinning apparatus. (**b**) Schematic of the continuous yarn. (**c**) SEM image of yarns after spinning. Reproduced with permission [80]. Copyright 2006, John Wiley and Sons.

Li et al. firstly proved that it is possible that spinning fibers of CNTs directly during the process of CVD [78]. On this basis, Vilatela et al. controlled the condition of the CVD and the rate of collector to prepare the fibers with high performance and shed light on the fact that the CNTs were oriented and parallel to the fiber axis in the process [79]. Zhang et al. reported a distinct method that using the super-aligned CNT arrays made by themselves achieved directly the fabrication of the yarns with aligned CNT, as shown in Figure 10 [80]. During the process of spinning, continuous yarns were produced due to the strong van der Waals force between CNTs. Apart from that, Gommans et al. generated continuous fibers with oriented CNTs from solution [81].

Obviously, the force-induced methods have huge advantages that available devices, simple operation and improvement of mechanical properties. However, deterioration of electrical and dielectric properties is usually produced during the process of alignment, and it is almost impossible to achieve accurate control of an individual CNT in this way.

### 3.2. Film Assisted Method

#### 3.2.1. Langmuir-Blodgett Film

**Figure 11:**
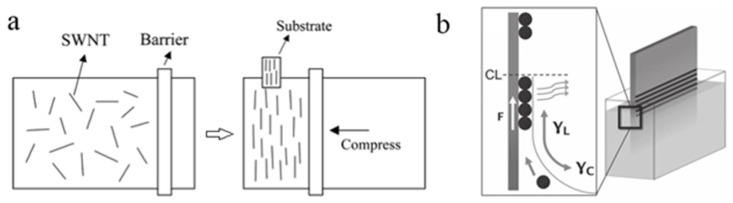
(**a**) Process of LB film technique. Reproduced with permission [84]. Copyright 2008, AIP Publishing. (**b**) schematic of the evaporation-driven assembly method. Reproduced with permission [85]. Copyright 2012, John Wiley and Sons.

Langmuir-Blodgett film (LB film) technology is used to fabricate monomolecular thin film which transfers from horizontal Langmuir monolayer to a vertical solid substrate. The first application of the LB film in one-dimensional nanomaterial is about the study of oriented nanorods [82]. Several years later, Li et al. applied this kind of technique in functionalized SWNTs to achieve large-scale assembly [83]. Lu et al. introduced a model to explain the compression-induced alignment during the process of forming LB film and prepared multilayers of SWNT LB film, as shown in Figure 11a [84]. Similar to LB film, evaporation-driven assembly also can be used to orient CNTs with an intermittently moving substrates, as shown in Figure 11b [85].

#### 3.2.2. Blown Bubble Film

**Figure 12:**
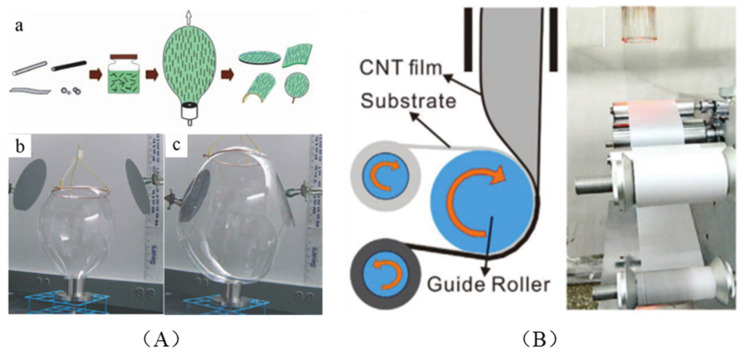
(**A**) (**a**) Schematic of blown bubble films method; (**b**,**c**) Pictures of the initial and final stage of blown bubble method. Reproduced with permission [86]. Copyright 2008, Royal Society of Chemistry. (**B**) Schematic of the apparatus and Photograph of collecting process. Reproduced with permission [44]. Copyright 2020, John Wiley and Sons.

Here, an interesting method is introduced that Cao et al. combined blown film extrusion and nanocomposite film for the first time [42]. Using a die to form a bubble from polymer suspension of CNTs with controlled blowing, they achieved the fabrication of large-area substrates with aligned CNTs. Then this approach also was applied to other nanomaterials and on flexible substrates with good alignment, as shown in Figure 12A [86]. After that, they continued to fabricate isotropic CNT film by a layer-by-layer transfer process [87]. Inspired by the blown bubble technique and the floating catalyst CVD, Xie et al. recently prepared the flexible transparent conductive films with excellent performance, as shown in Figure 12B [44].

Film-assisted methods can achieve large-scale alignment of CNT easily, so it is feasible to realize commercial applications in this way. Another advantage is that this approach can be employed for flexible films and devices which have broad impact and huge potential on the number of fields. However, it is impossible to manipulate individual CNT by using this pathway.

### 3.3. Electric Field Method

#### 3.3.1. Electric Field Assisted Method

**Figure 13:**
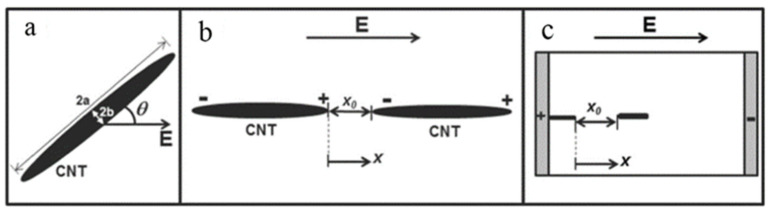
Schematics of the three dynamic mechanisms of CNT under applied AC electric fields: (**a**) CNT rotation, (**b**) Coulombic interaction between CNTs, (**c**) CNT migration towards an electrode. Reproduced with permission [89]. Copyright 2014, Elsevier.

**Figure 14:**
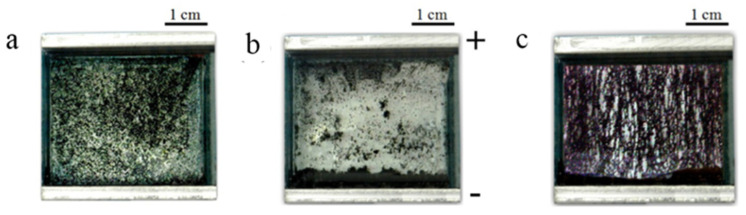
Optical photographs of MWCNT in distill water under an applied electric field. (**a**) V = 0 V, (**b**) V_DC_ = 200 V after 120 s, (**c**) V_AC_ = 400 V at 1 kHz after 5 s. Reproduced with permission [96]. Copyright 2012, IOP Publishing.

In 1996, Fishbine originally discovered that CNTs can be electrically polarized in electrostatic fields, which induced the electrostatic dipole moment to achieve the alignment of CNT [88]. On this basis, Oliva et al. proposed a modeling of the dynamic carbon nanotube network under alternating current electric fields to systematically elucidate the process of CNT migration and three dynamic mechanisms including CNT rotation induced by electrostatic dipole moment, Coulombic interaction between CNTs and CNT migration towards an electrode induced by dielectrophoresis force as shown in Figure 13 [89]. Banda et al. introduced the efficient dispersion of CNTs in the polymer in detail and demonstrated the improvement of electrical and mechanical properties after alignment [90]. It also was found that orientation of CNT can be achieved in both AC [90,91,92,93,94,95,96,97] and DC [92,96,98,99,100]. electric fields as shown in Figure 14, and more uniform and aligned network structures can be achieved in the AC electric field than in the DC electric field [92]. Besides that, field magnitude [90], Concentration [95,99], frequency [96] and required time [101] are key factors in the alignment of CNT.

Liquid solutions and polymers are common mediums for aligning CNT in electric fields. It has been proved that CNTs can rotate and eventually orient along the direction of electric fields in various liquid solutions, including isopropyl alcohol [91], distilled water [97] and ethanol [98]. Park et al. achieved the alignment of SWNTs in a photopolymerizable monomer solution immobilized by photopolymerization under a continuously applied AC electric field [93]. Oliva et al. fabricated CNT/polymer composite films with aligned CNTs under the AC field [95]. Chapkin et al. monitored the arrangement of CNTs under an applied electric field in situ, real time by a polarized Raman spectroscopy, which can assist in determining processing condition [101]. Owing to the lower viscosity and the higher permittivity of liquid solutions than polymers resulting in lower damping term and higher dipole moment, CNTs can achieve alignment in a shorter time [95]. 

#### 3.3.2. Electrospinning 

**Figure 15:**
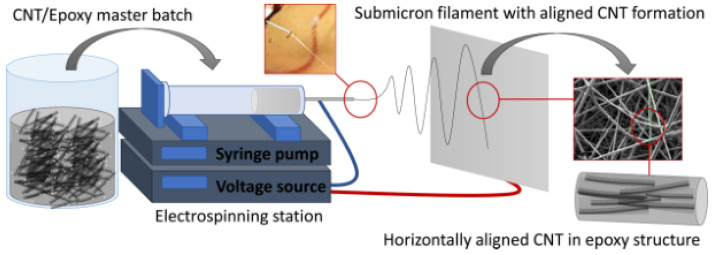
Precess of electrospinning for filament with aligned CNT formation. Reproduced with permission [41]. Copyright 2021, American Chemical Society.

Electric fields can be combined with other technologies for aligning CNT, and electrospinning is a typical example. Electrospinning, generally, is another a simple and common method for drawing nanoscale fibers from polymer solution or melt [102]. To improve the properties of fibers, incorporating CNTs in fibers emerged to prepare CNT-polymer composites and provided a new means for CNT alignment. A theory was presented to explain the alignment of CNT in electrospinning. Due to the flow in a wedge, random CNT are oriented along the nozzle gradually [103]. Frank et al. prepared PAN fibers containing aligned SWNT by electrospinning, and found that the alignment of SWNT in PAN fibers (50–200 nm) is better than in PLA fibers (1 mm) [104]. Sen et al. also fabricated the PU nanofibers and membranes with aligned SWNT by electrospinning, which exhibited a significant enhancement in the mechanical properties [105]. Hou et al. prepared CNT/PAN composite nanofiber sheets by the electrospinning with the moving collector which aligned nanofibers on nanofiber sheets [106]. Although there still are some obstacles in the electrospinning of thermosetting fibers, recently, Aliahmad et al. fabricated thermosetting fibers embedded in aligned CNTs networks, as shown in Figure 15 [41]. 

**Figure 16:**
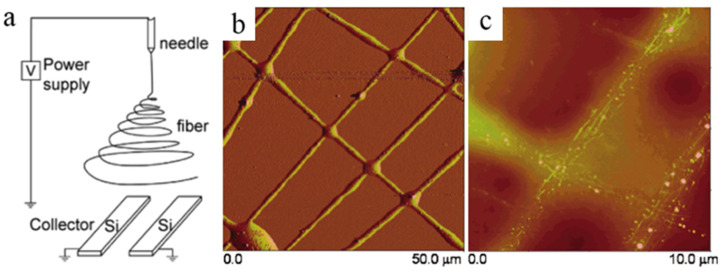
(**a**) Schematic of the collector with two pieces of conductive silicon strips separated by a gap. Reproduced with permission [107]. Copyright 2003, American Chemical Society. AFM images of (**b**) cross-aligned SWNTs/PVP composite fibers and (**c**) cross-aligned SWNT array after etching PVP. Reproduced with permission [108]. Copyright 2004, American Chemical Society.

Li et al. proposed an original approach that the collector contained two pieces of conductive silicon strips separated by a gap as shown in Figure 16a [107]. During the process of electrospinning, the charged nanofibers were stretched to span across the gap, to form uniaxially aligned arrays. Influenced by this idea, Haddon et al. prepared SWNTs composite fibers, in which CNTs aligned along the axial direction of polymer-CNTs composite fibers as shown in Figure 16b [108]. In the experiment, they chose PVP as basal material that has a good compatibility with SWNTs. Under the action of static electricity, they directly obtained the oriented polymer-carbon nanotubes composite fiber by electrospinning. The polymer on the surface of SWNTs was etched to obtain the oriented CNTs array. Zhang et al. continued to complete this method by improving the collector, which is a rotating drum with parallel copper wires seen as electrodes [17]. In addition, there are other types of collectors modified to collect aligned CNTs during the process of electrospinning, such as auxiliary electrode collector [109], high-speed rotating collector [110], ring collector [111] and wire spring collector [112]. 

In addition to electrospinning, electric fields can be combined with other technologies, such as the synthesis of in site CNTs [113] and 3D printing [114]. 

As mentioned above, the electric field approach is available and can be applied to both solution and polymer, which means that it can be easily combined with other technologies. Another advantage is that CNTs can achieve oriented alignment without contact in this way, which ensures the performance of CNT. To a certain extent, an individual CNT can be manipulated by dielectrophoresis forces to fabricate microelectronic devices. As continuous improvement, Electrospinning technology can be used to realize the large-scale manufacture of fibers with aligned CNTs. 

### 3.4. Magnetic Field Method

**Figure 17:**
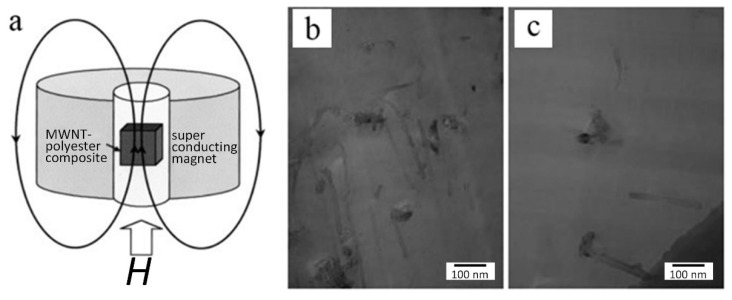
(**a**) Schematic of the alignment of CNTs in composite under magnetic field. TEM image of a thin film sliced (**b**) parallel and (**c**) perpendicular to the applied magnetic field. Reproduced with permission [116]. Copyright 2002, John Wiley and Sons.

Similar to the electric field method, the magnetic field also can be used to align CNTs without contact. Walters introduced the suspension of SWNTs to a strong magnetic field of 19T to align segments and then achieved the preparation of thin membranes [115]. As shown in Figure 17, Kimura et al. placed MWNT polyester composite in a mold surrounded by a constant magnetic field of 10T and verified the ordered result by measuring magnetic susceptibility, conductivity, and elastic modulus [116]. Not only can the high magnetic field achieve orientation, Ma achieved the alignment of CNTs in composite under a low magnetic field of 0.4T, which has a significant enhancement in toughness and fracture energy [117]. With respect to the alignment of CNTs in solution, Bhardwaj et al. poured the solution mixed by CNTs and polymer on glass plates kept under magnetic field [118]. After drying overnight, the CNTs in these polymer films aligned along the direction of the magnetic field, which could be measured with the help of Raman spectroscopy. 

Magnetic field can be applied into other techniques as same as electric field. Lee et al. achieved the control of the growth direction of CNTs correlated with the direction of the magnetic direction using CVD technique [119]. Yang et al. chosen two parallel magnets as the collectors of the electrospinning, which generated the well-aligned fibers parallel along the magnetic field lines [120]. 

In general, magnetic field has many same advantages as electric field, such as non-contact, availability and flexibility. However, due to the absence of dielectrophoresis force, the movement and manipulation of individual CNT is impossible.

### 3.5. Printing Method

#### 3.5.1. Direct Writing Printing

**Figure 18:**
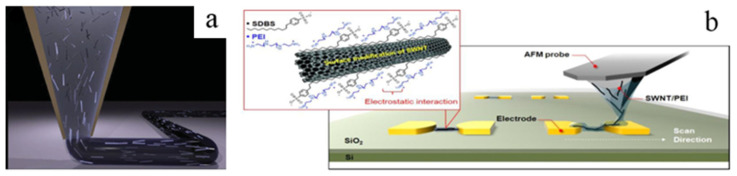
(**a**) Schematic of the alignment of high aspect ratio fillers during the process of extrusion. Reproduced with permission [122]. Copyright 2014, John Wiley and Sons. (**b**) Schematic of patterning SWNTs using the direct-write dip-pen nanolithography approach. Reproduced with permission [125]. Copyright 2016, American Chemical Society.

Figure 18a illustrates the fact that the high aspect ratio filler induced by the force of shear and extrusion align along the printing direction in the process of direct writing printing [121,122]. Lewicki et al., firstly, reported an example of additively manufactured carbon fiber composite materials through an adaptation of direct ink writing 3D-pringing technology [123]. In the experiment, the carbon fiber phase in the ink transferred from a random orientation to an ordered alignment on the effect of the microextrusion in the print head inducing shear alignment. Farahani et al. designed and fabricated two strain sensors made of SWNT composites with a fairly high electromechanical sensitivity by direct writing printing assisted by the ultraviolet used to accelerate the curing of the materials printed, which is a novel way to manufacture microelectronic devices [124]. Lee at al. proposed a new method of dip-pen nanolithography in which the tip of an atomic force microscope acted as a “pen” coated with a composite containing SWNTs with the advantage of the non-destruction as shown in Figure 18b [125].

Zhou et al. reported a method of stereolithography with a pair of rotatable electrodes controlling the direction of MWNT during the printing [114]. They used the mixture of surface modification of MWNT and polymer resin as printing material and built a complex architecture with different mechanical property controlled by the orientation of CNTs. 

#### 3.5.2. Inkjet Printing

**Figure 19:**
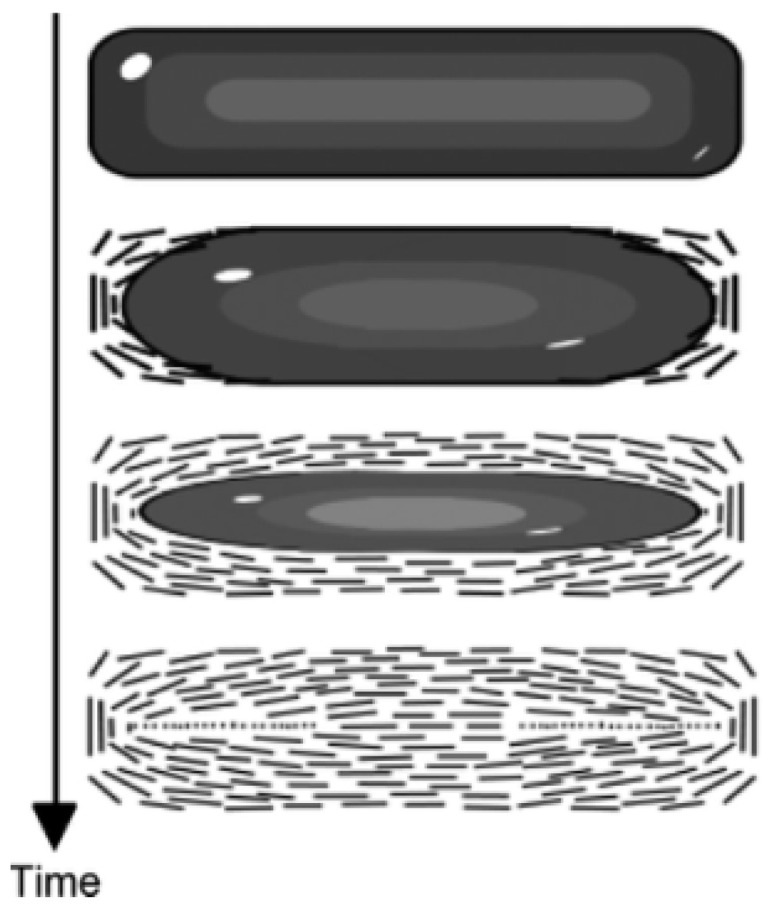
Process of evaporation behavior of an inkjet printed line resulting in oriented CNTs. Reproduced with permission [126]. Copyright 2012, American Chemical Society.

Utilizing the intrinsic liquid crystal behavior and “coffee ring” effect, Beyer et al. prepared films of highly aligned CNTs by controlling the deposition and evaporation rates during inkjet printing, as shown in Figure 19 [126]. And the lines generated in inkjet printing are quite thin, so the CNTs can be regarded as highly aligned.

In 2006, Kordas et al. reported a typical study about generating CNTs patterns using a commercial desktop inkjet printer. After that, there have been a number of research studies into CNT inkjet printing [127]. Song et al. investigated the influences of the experiment conditions, including substrate heating, surface hydrophilicity and jetting process and the electrical properties of the sprinted lines with different linewidths and printing times [128]. Okimoto et al. prepared the printable solution and achieved the manufacture of CNT thin-film transistors, which exhibit better performance than conventional transistors [129].

To date, inkjet printing technique has been applied in many electronic devices, such as sensors and transistors. Still, the preparation of the ink meets some bottlenecks, such as relatively complex process and poor applicability. With the further research for the ink, it is generally believed that the combination of inkjet printing and CNTs will bring exciting performance in the near future. 

### 3.6. Other Methods

In addition to the methods mentioned above, there also are many researches on alignment of CNT utilizing the approaches such as self-assembly [130,131], liquid crystals [132,133], surface acoustic waves [134].

## 4. Summary

On the pathway of successful application and commercialization of CNT, ordered alignment is a tough but inevitable step, which needs more research into the mechanism and condition to meet what people expect. In this review, we summarize the main approaches for aligning CNT from two aspects of vertical and horizontal alignment and attempt to give the advantages and disadvantages of these approaches. But it is hard to say that which method is best; they can exhibit different edges in different application scenarios. And there is no doubt that creating a clear boundary between different categories is almost impractical; some approaches integrate several categories. Nevertheless, it is still beneficial to help people understand the arrangement of CNTs. 

It is obvious that there are more and more approaches to achieve alignment of CNT, which bring more possibilities to both the manufacture and application. The CNTs/polymer composite with the aligned CNTs can further enhance the performance of mechanical, thermal and electrical along the CNTs axial direction, which can be used in sport equipment, electrostatic dissipation and electromagnetic interference shielding [135]. with the aspect of electrodes, aligned CNTs electrodes have higher specific capacitance, lower equivalent series resistance and better rate capability than unordered CNTs electrodes, which can be used in lithium-ion battery, solar cells and so on [136]. The well-aligned CNT arrays also could have the more possibility in electronic and microelectronic, which can be used to prepare top-gate field-effect transistors with better performance than commercial silicon metal oxide-semiconductor field-effect transistors [137]. At the same time, the combination of different methods also brings more potential.

Besides the macroscale alignment of CNT, with the rapid development of nanotechnology, the microscale alignment and manipulation give a new direction and have huge development potential. By using a hybrid atomic force microscope and scanning electron microscope system, the precise placement of a single CNT can be achieved onto a microelectromechanical system [138]. Dielectrophoresis can also be used to manipulate individual or multiple CNT to bridge electronic conductors [139].

The aligned CNTs can further improve the performance, broaden the application fields, and bring us more surprises. Although it is still mainly studied in the laboratory now, it will appear in our side in the near future. We believe that this paper will broaden the research strategies and contribute to the wide range of application of CNT.
